# Review of computational neuroaesthetics: bridging the gap between neuroaesthetics and computer science

**DOI:** 10.1186/s40708-020-00118-w

**Published:** 2020-11-16

**Authors:** Rui Li, Junsong Zhang

**Affiliations:** 1 National Engineering Laboratory for Educational Big Data, Central China Normal University, Wuhan, Hubei People’s Republic of China; 2 Fujian Key Laboratory of Brain-Inspired Computing Technique and Applications, Department of Artificial Intelligence, School of Informatics, Xiamen University, Xiamen, China

**Keywords:** Neuroaesthetics, Computational aesthetics, Computational neuroaesthetics, Brain functional connectivity, Machine learning

## Abstract

The mystery of aesthetics attracts scientists from various research fields. The topic of aesthetics, in combination with other disciplines such as neuroscience and computer science, has brought out the burgeoning fields of neuroaesthetics and computational aesthetics within less than two decades. Despite profound findings are carried out by experimental approaches in neuroaesthetics and by machine learning algorithms in computational neuroaesthetics, these two fields cannot be easily combined to benefit from each other and findings from each field are isolated. Computational neuroaesthetics, which inherits computational approaches from computational aesthetics and experimental approaches from neuroaesthetics, seems to be promising to bridge the gap between neuroaesthetics and computational aesthetics. Here, we review theoretical models and neuroimaging findings about brain activity in neuroaesthetics. Then machine learning algorithms and computational models in computational aesthetics are enumerated. Finally, we introduce studies in computational neuroaesthetics which combine computational models with neuroimaging data to analyze brain connectivity during aesthetic appreciation or give a prediction on aesthetic preference. This paper outlines the rich potential for computational neuroaesthetics to take advantages from both neuroaesthetics and computational aesthetics. We conclude by discussing some of the challenges and potential prospects in computational neuroaesthetics, and highlight issues for future consideration.

## Introduction

Aesthetics, defined in the dictionary as “a set of principles concerned with the nature and appreciation of beauty” [1], plays a fundamental role in human’s history and culture. The way we appreciate beauty and the way we create beautiful things enrich our daily life and fulfill our world. However, we still know little about how aesthetics, the feeling of being moved and the ability to appreciate and judge the beauty, is generated. Aesthetics has been a subject of curiosity within philosophy in the eighteenth century, but now has extended to other scientific disciplines like cognitive psychology, neuroscience, and computer science [2–4]. In these fields, researchers seek to understand aesthetics from different perspective. The widespread interest on aesthetics and the integration of aesthetics with researcher’s own background have brought out a variety of interdisciplinary research fields such as empirical aesthetics, neuroaesthetics, and computational aesthetics [5, 6].

Empirical aesthetics has emerged from the field of experimental psychology in the 19th century, where Gustav Fechner was attracted by the mystery of aesthetics. He developed the initial methodological setup to carry out experimental research on aesthetic experience [7]. After that, numerous experimental researches on aesthetics have been carried out by psychologists who seek to understand the psychological process during aesthetic appreciation and creation. In empirical aesthetics, results are usually obtained by the observation of behavior and questionnaire from participants in well-designed experiments. Findings from the experimental results uncovere a series of psychological factors such as perception, knowledge, and content, which influence our aesthetic experience of art. However, these findings mainly focuse on building theoretical models of aesthetics and they are insufficient to fully reveal the mystery of aesthetics. The lack of support by the neural basis of aesthetic experience and quantitative computing between different psychological factors make aesthetics remains a mirage. Neurobiological investigation started by neuroscientists like Cajal (1852–1934) builds our basic understanding of the neural basis of human behavior [8]. With the advance of technology, modern neuroimaging tools, such as electroencephalogram (EEG), magnetoencephalogram (MEG), and functional magnetic resonance imaging (fMRI), have become available for researchers. The application of these neuroimaging techniques in experimental aesthetics and the goal to understand neurobiological basis of our cognitive process during aesthetic experience have blossomed into a newly research perspective called neuroaesthetics [9]. This research field grows fast and important findings in neuroaesthetics continue to enhance our understanding of neural underpinning of aesthetics.

While the burgeoning neuroaesthetics becomes a research enterprise, another field, computational aesthetics has also gain increasing interest from researchers. Computational aesthetics is defined as “the research of computational methods that can make applicable aesthetic decision in a similar fashion as humans can” [6]. The idea of computational aesthetics can be dated back to Fechner who believed that the physical attributes in aesthetic appealing things could be measured in a formalistic way. Then, in 1933, Birkhoff wrote the book entitled “Aesthetic Measure” and proposed a formula to measure aesthetics in a very mathematical way [10]. His work is often regarded as the beginning of computational aesthetics. Heavy use of computers in the modern information society and prevalence of aesthetics in our daily life have led to motivation for researches in computational aesthetics. As a subfield of computer vision, computational aesthetics seeks to build computational models to give an aesthetic evaluation on visual stimuli or generate art like professional artists automatically. The wide application of computational aesthetics in image assessment as well as art generation makes it a hot topic in recent years [11]. Studies in computational aesthetics not only can be the test bed for aesthetic measurements but also stretch our understanding on visual attributes that affect aesthetic appreciation and art generation.

Both neuroaesthetics and computational aesthetics can enrich our understanding on aesthetic appreciation. And these two fields can benefit from each other by proposing new theoretical models as well as computational models. However, there is still a gap between neuroaesthetics and computational aesthetics. Theoretical findings from neuroaesthetics cannot be immediately formulized in computational aesthetics and some computational models from computational aesthetics lack of experimental validation and theoretical support by neuroaesthetics. With the development of neuroaesthetics and computational aesthetics in nowadays, to give a comprehensive understanding on aesthetics, it is time to integrate findings from the two fields and bridge the gap between them.

**Fig. 1:**
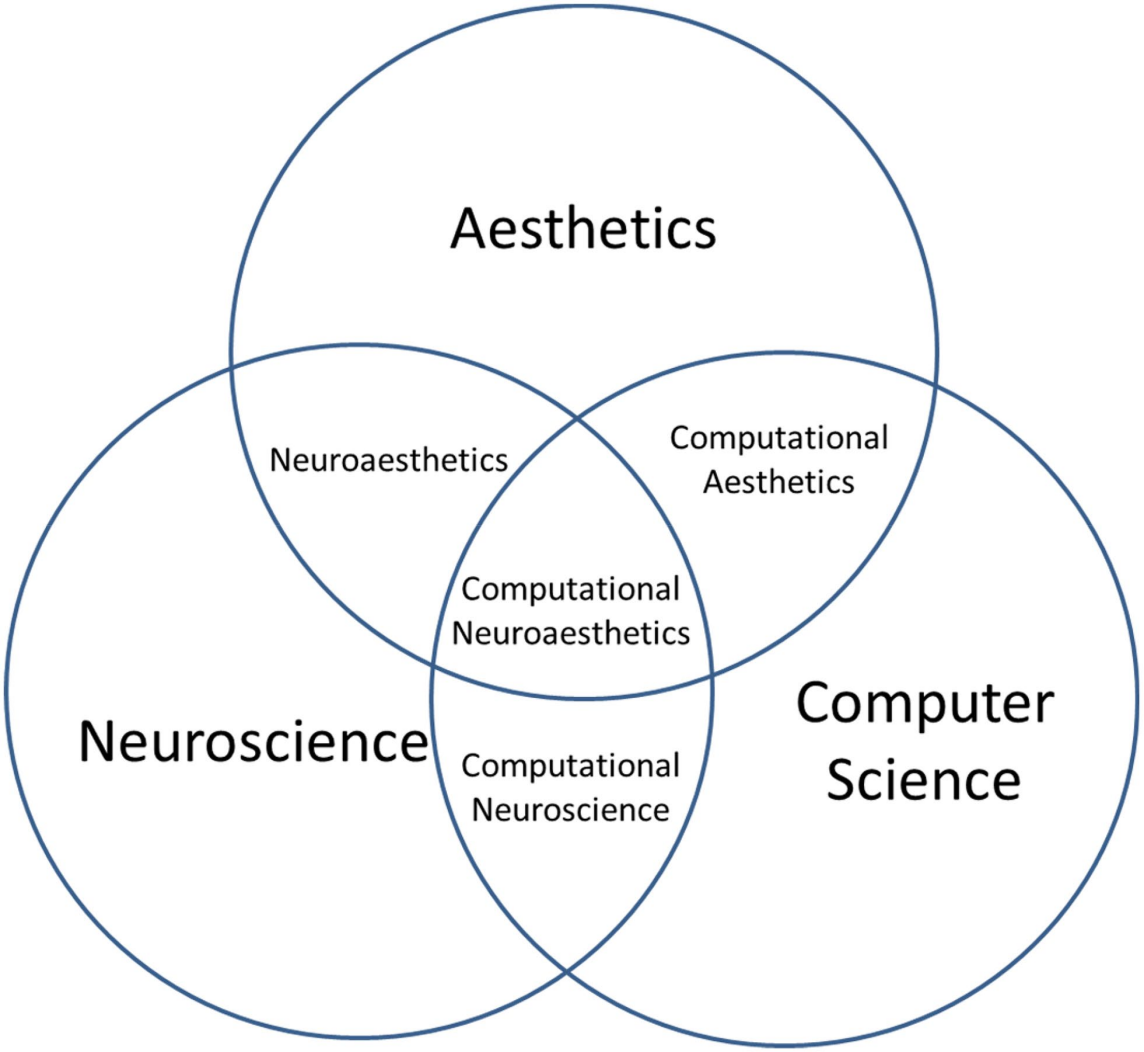
Computational neuroaesthetics has emerged from three interdisciplinary fields: Neuroaesthetics which combines neuroscience and aesthetics, computational aesthetics which employs computer science to investigate aesthetics, and computational neuroscience which uses mathematical tools and theories to investigate brain function

Computational neuroaesthetics, although still in its infancy, seems to be promising to assemble the pieces of puzzle from neuroaesthetics and computational aesthetics. It has emerged from three research fields including aesthetics, neuroscience, and computer science. It roots in neuroaesthetics as well as computational aesthetics. And its concept of understanding and modeling neurobiological components in aesthetics by computers is partially come from computational neuroscience (see Fig. 1). Studies measuring brain information flows during aesthetic appreciation and developing computational models based on neural activities to make aesthetic evaluation predictions should be central to computational neuroaesthetics.

In our paper, we briefly reviewed the developments in both neuroaesthetics and computational aesthetics. Then, we introduced findings and challenges in computational neuroaesthetics. Our goal is to gain interest from researchers in both neuroaesthetics and computational aesthetics to the findings and opportunities in computational neuroaesthetics. Since computational neuroaesthetics is far from mature, studies within its scope should be encouraged and wealth findings in neuroaesthetics and computational aesthetics could be fertilizer to it. Specifically, our review mainly focuses on aesthetic appreciation and aesthetic experience of visual images. Topics include theoretical models and neural underpinning of aesthetic appreciation from neuroaesthetics, automatic image quality assessment by computers from computational aesthetics, and finally, computational models for measuring and modeling brain activities in computational neuroaesthetics.

## Developments in neuroaesthetics and computational aesthetics

Neuroaesthetics is emerged as an independent discipline which investigates biological bases of aesthetic experience when we appraise objects [12]. Its emergence is mainly attributed to the pioneering work from vision researcher S. Zeki. After 20 years of development, this nascent field has gained more and more interest from researchers in psychology and neuroscience. Numerous experiments are carried out to illustrate neural mechanism of aesthetic appreciation and theoretical models have been proposed to outline cognitive process of the cascading timelines of aesthetic experience. Also, there is a range of literatures written by experts in this field to review and discuss previous findings in neuroaesthetics [13–19]. Hence, we briefly introduce important conceptual models and neural systems related to aesthetic appreciation.

### Conceptual models

#### Zeki’s model and Ramachandran and Hirstein’s model

Early models mainly concentrated on the visual art and its physical properties which reflected in our brain. In the 20th century, Zeki reviewed thoughts from previous philosophers, neurologists, and artists. Based on their views, he proposed that the function of art is an extension of the visual brain: a set of parallel processing-perceptual systems that distill important information which represents essential characteristics of objects from the ever-changing visual world and build up true knowledge [9, 20]. Zeki’s model links aesthetics to biological functions of the brain. And it explains perception of visual art and its relationship with visual areas in the brain. Yet, an explicit description about the intervention of cognitive functions during aesthetic experience seems not to be given in Zeki’s model.

Later, Ramachandran and Hirstein combined evolutionary approach with neurophysiological evidence and proposed a model to explain aesthetic experience on visual art [21]. This model includes several principles elaborating how brain processes arts by applying a reinforcement mechanism: first, visual objects are discriminated by peak shift effect; then, features are extracted and grouped into unitary clusters by different visual areas; a certain feature which is special importance is reinforced by activation of both limbic structures and allocation of attentional resources to produce pleasurable rewarding sensations. Their model is more elaborated than Zeki’s model, although the cognition and emotion during aesthetic experience remain elusive.

#### Leder’s model

In 2004, Leder proposed a visual aesthetic model based on previous findings in psychology and neuroscience, and this model was improved in 2014 [2, 22]. Following Leder’s model, information processing of aesthetic appreciation is divided into five stages: 1) Context and input of the model: visual stimulus first undergoes a pre-classification stage in which stimulus is estimated whether it can trigger interest and emotional response. 2) Perceptual analysis: in this stage, physical attributes of stimulus such as complexity, contrast, symmetry, and combination are perceived and analyzed. 3) Implicit memory integration: analysis results from perception of stimulus are combined with implicit memory effects such as familiarity, prototypicality, and other information to give an implicit aesthetic judgment. 4) Explicit classification: this stage includes discrimination of the type and content of stimulus. And cognitive processing is affected by the expertise and knowledge of perceiver to give an explicit classification. 5) Cognitive mastering and evaluation: this stage includes explaining stimulus from an aesthetic point of view and combining stimulus with self-related cognitive information to achieve a successful evaluation. In Leder’s model, aesthetic evaluation of stimulus includes both cognitive mastering of aesthetic properties and satisfaction of emotional state. The former one eventually leads to aesthetic judgment, while the later one triggers aesthetic emotion.

#### Chatterjee’s model

In 2003, Chatterjee has proposed a linear processing model on visual aesthetic experience [3]. In his model, visual aesthetic experience includes three cognitive processing stages: First, early visual processing which takes place in different brain regions extracts information from visual object and simple components are analyzed. Second, pre-processed components are decomposed and integrated to form a coherent embodiment. Finally, particular brain regions are activated to further analyze the embodied elements. In 2014, Chatterjee and Vartanian reviewed previous evidence in neuroaesthetics and improved their theoretical model [5]. In the recent model, aesthetic experience emerges from the interaction of three neural systems: sensory–motor system, emotion–valuation system, and knowledge-meaning system. This model is useful to connect various aspects of cognitive processing stages of aesthetic experience to particular brain structures.

#### Redies’ model

Redies proposed a model of aesthetic experience for the cognitive process on visual pictures [23]. In his model, stimulus acquires its form from a visual aspect, which is necessary for sensory perception and content information. The sensory input triggers perceptual processing, which causes an emotional reaction and generates an aesthetic of perception. Content information, however, passes through cognitive communication and requires a combination of memory retrieval to generate a cognitive processing that produces aesthetic cognition for an aesthetic experience. In Redies’ model, aesthetic perception is a fast, bottom–up, universal process; aesthetic cognition is a top–down, cultural, individual process that is more associated with the personal experience of beauty appreciation.

As we have seen, a number of theoretical models have been proposed. These models try to explain cognitive process of aesthetic appreciation from different perspective and give a concept on factors which may influence aesthetic appreciation. However, these models are usually unique on their hypotheses and interpretations which make them incompatible with each other. Thus, it is hard to combine these models and integrate components within them to give a comprehensive understanding. Furthermore, some models are well suit for holding the evidence from neuroaesthetics. Other models based on empirical studies are difficult to illustrate hypotheses about specific brain activity, but give a conceptual guideline. To take a deep look at aesthetic appreciation, theoretical models should be combined with the evidence of brain activities during aesthetic appreciation and these models need to be complemented when new findings on neural mechanism of aesthetic appreciation are uncovered [24, 25].

### Activity of brain regions during aesthetic appreciation

**Fig. 2:**
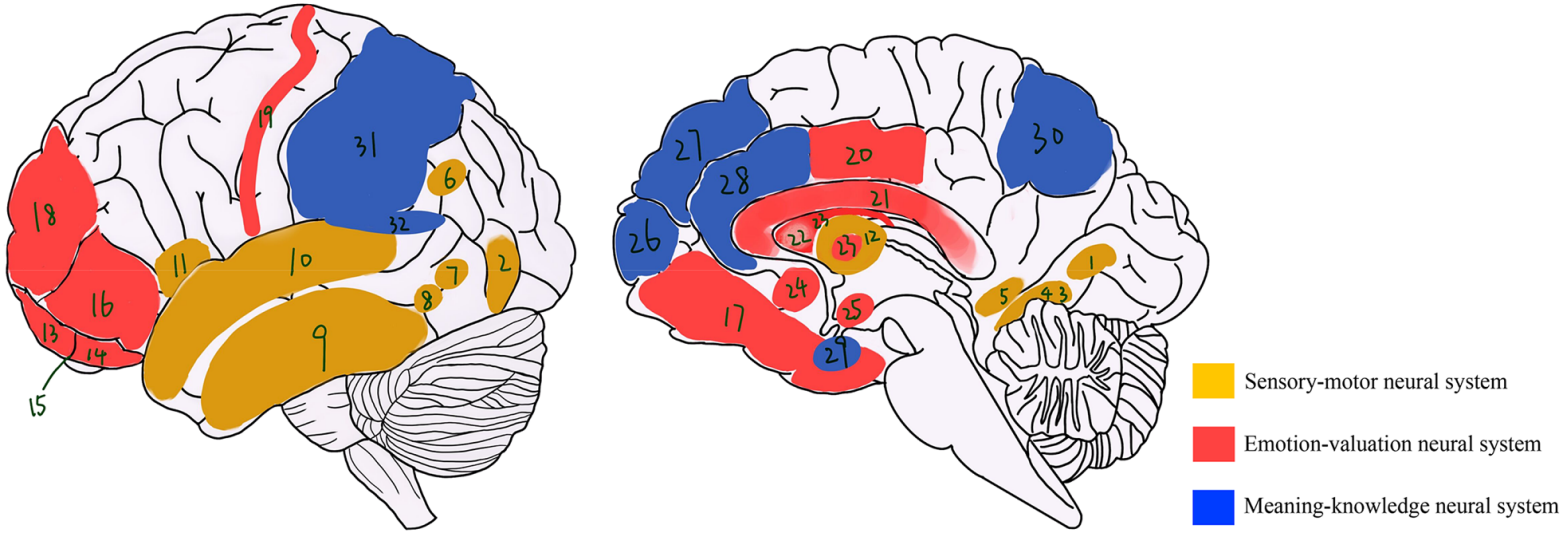
Brain regions from three neural systems. Sensory–motor neural system includes brain regions marked with yellow color: 1. lingual gyrus, 2. middle occipital gyrus, 3. fusiform face area, 4. fusiform gyrus, 5. parahippocampal gyrus, 6. bilateral angular gyrus, 7. visual motion area, 8. extrastriate body area, 9. inferior temporal cortex, 10. superior temporal gyrus, 11. anterior insula bilaterally, and 12. putamen. Emotion–valuation neural system includes brain regions marked with red color: 13. medial OFC, 14. lateral OFC, 15. OFC, 16. VLPFC, 17. VMPFC, 18. DLPFC, 19. fronto-temporal junction, 20. Posterior cingulate cortex, 21. caudate nucleus, 22. ventral striatum, 23. striatum, 24. nucleus accumbens, and 25. amygdala. Meaning-knowledge neural system includes brain regions marked with blue color: 26. Brodmann areas 9/10, 27. dorso-medial prefrontal cortex, 28. bilateral anterior cingulate cortex, 29. temporal pole and entorhinal cortex, 30. precuneus, 31. superior and inferior parietal cortex, and 32. temporoparietal junction

Previous neuroimaging studies have found that several brain regions and their functionalities are essential in constituting our aesthetic experience [17, 19, 26]. During aesthetic appreciation, these brain regions are activated to process physical attributes of art, link the content to the knowledge, and finally give an aesthetic judgement. Investigating engagement of brain regions during aesthetic appreciation can unravel the neural mechanism of aesthetic experience. Neuroimaging studies on the activity of brain regions, like puzzles, together with theoretical models provide us insight to the blueprint of neuroaesthetics. Among the existing theoretical models, Chatterjee’s model is directly grounded on the evidence from neuroaesthetics, which makes it a good candidate to integrate findings of brain activity to explain phenomena central to aesthetics. According to Chatterjee’s model in 2014, aesthetic experience emerges from the interaction of three neural systems. Each neural system includes several brain regions introduced as below (see Fig. 2).

#### Sensory–motor neural system

The sensory–motor neural system underlies sensation and perception of aesthetic appreciation. This neural system contains several brain regions in visual and motor areas. Brain regions in the visual area support the function of searching for features from objects, scenes, and people during observation of different paintings. Among these brain regions, the lingual gyrus and the middle occipital gyrus are activated when processing various early, intermediate and late visual features of paintings such as orientation, shape, color, grouping, etc. [27]. According to Luo’s study, the activation of lingual gyrus is associated with aesthetic appreciation of moral beauty and the middle occipital gyrus is activated when appreciating facial beauty [28]. Another brain region, the bilateral angular gyrus, is also activated when processing visual features. But it is specific on spatial processing strategies such as forms, color, symmetry, and complexity [29, 30]. This brain region demonstrates greater activation when processing beautiful stimuli. And it helps connect and integrate perception with attention, spatial cognition, and episodic memory. The inferior temporal cortex is regarded as an important brain region in processing visual representation of form and color while viewing paintings as well [31]. From the previous studies on paintings rich in representations of scene, the parahippocampal gyrus is activated when viewing scenes correlated with pleasure, suggesting that the parahippocampal gyrus is involved in the perception and recognition of specific stimuli such as places [32, 33].

The fusiform gyrus, according to previous studies, is correlated with aesthetic ratings and its activation seems to represent detection of visual objects like faces and bodies in paintings [34, 35]. In the fusiform gyrus, the fusiform body area is activated during the configuration of body and fusiform face area is activated when viewing attractive faces [36–38]. When viewing dynamic paintings and body movement art such as dance and calligraphy writing, the visual motion area, the extended mirror neuron system, and the extrastriate body area are activated. The visual motion area evokes subjective sense of movement [39]. The extrastriate body area is associated with viewing pictures or movies with legs, arms, or bodies in them. And the mirror neuron system, which is engaged in the processing of perception on artistic gestures and consequences of actions, represents an embodied element mirroring the movement and emotion expressed in artwork [40]. This brain region is crucial to understand the processing of our empathetic responses and engagement of emotional circuit to visual art [41, 42]. To process rewarding properties expressed in visual art, two brain regions, the putamen and bilateral anterior insula, are also engaged [43]. Specifically, the putamen is reliably activated when we anticipate rewards.

#### Emotion–valuation neural system

The emotion–valuation neural system underlies emotions introduced by evaluation of aesthetic objects. It includes several brain regions associated with the processing of reward and emotion. The frontal cortex, the frontal–temporal junction, and the orbitofrontal cortex (OFC) are involved in the evaluative judgment of affective components [44]. Among the three brain regions, the OFC gains great interest from researchers and profusely mentioned in neuroaesthetic studies. This brain region is an important area for experience and judgments of beauty [45, 46]. According to the studies on the OFC, different modalities, including visual aesthetics, auditory aesthetics, olfactory aesthetics, gustatory aesthetics, and moral beauty, are processed by the OFC, and each separate modality is processed in different areas of the OFC [14, 47, 48]. These studies suggested that the OFC involved in representing the reward value of stimulus irrespective of modalities of beauty. The OFC contains two subdivisions: the media OFC and the lateral OFC. From an interesting study performed by Ishizu and Zeki on investigating sorrowful and joyful photos, the OFC had a weaker response on sorrowful beauty than joyful beauty, while the media OFC was activated by both sorrow and joyful beauties [49]. Indeed, the media OFC have its domain specificity during aesthetic appreciation. According to the previous studies, this brain region is associated with the experience of reward and emotion. And it co-activated with the visual/auditory sensory and perceptual areas. In Tsukiura’s study, both beautiful faces and beautiful moral actions activated the media OFC [46]. And in Zeki’s study, higher score when participants were rating music excerpt was accompanied by stronger activation of the OFC [50]. Interestingly, by another study from Zeki et al., the media OFC was activated when mathematicians rate the mathematical formulas as beautiful; suggesting that it is related to engagement with beautiful ideas [51]. The lateral OFC, as another subdivision of the OFC, is heavily connected with the media OFC. This brain region is found to be selectively activated for facial attractiveness [52]. From the previous studies of aesthetic experience, the media OFC tends to be a major area, while the lateral OFC is related to punishment or overriding of rewarded stimuli [53].

Along with the OFC, the ventrolateral prefrontal cortex (VLPFC), the dorsolateral prefrontal cortex (DLPFC), and the ventromedial prefrontal cortex (VMPFC) constitute the prefrontal cortex. The VLPFC is considered anatomically synonymous with the OFC, but it has distinct neural connections and performs distinct functionality. From previous studies on aesthetic appreciation, this brain region is supposed to be associated with superior attentional loads and it is probably mediated by thalamus [45, 54]. The DLPFC is activated in moral beauty condition and when decision takes place [15]. It acts as an integrator of signals coming from different visual sources and is associated with inherent aesthetic judgment. Specifically, the left DLPFC is involved in aesthetic experience as a center linking perception and action in multiple brain functions [55–57]. The VMPFC is supposed to be involved in the experience of reward value and acts as a common currency for preference [52]. It is activated when viewing attractive face, when processing the scene of nature, and during the experience of moral beauty [58]. When viewing both face and place attractiveness, posterior and ventral subregions of the VMPFC exhibit domain-specific activity [52]. And the media prefrontal cortex, parts of the VMPFC, is involved in many cognitive processing like autobiographical memory, experience of positive emotion, and decision-making about self.

Other brain regions widely distributed in brain are also related to emotion–valuation during cognitive processing of aesthetic appreciation. The right anterior insula, a brain region originally deployed for the purpose of survival advantage, is found to be associated with visceral perception and experience of emotions. This brain region is in brain’s core affective system and processes four forms (visual, auditory, gustatory, and olfactory) of beauty [59]. The bilateral insula is activated when subjects perform aesthetic orientation than pragmatic orientation [56]. Other study suggested that it may be related to the processing of eudemonic pleasures [60]. The striatum, the amygdala, and the nucleus accumbens are suggested to be associated with hedonic pleasures according to previous studies. The striatum integrates perceptual, evaluative, and reward components of aesthetic response irrespective of the modality and gives an aesthetic judgment [45]. The amygdala is thought to represent a subjective emotional response for experience of beauty according to the studies on sculptures and music [61]. The nucleus accumbens is important in reward system and influences our experience of pleasure [62]. The ventral striatum is activated by attractive faces and engaged in coding the reward probability [45, 63, 64]. Activity of the posterior cingulate cortex is related to memory retrieval and familiarity [54]. And the caudate nucleus is involved in many aspects of our experience of reward and plays a general role in evaluative judgment [44, 57].

#### Meaning-knowledge neural system

The meaning-knowledge neural system contains several brain regions related to the processing of context. The contribution of this neural system is less studied by researchers than sensory–motor and emotion–valuation neural systems. Partly because of its manifestations are widely distributed throughout the brain and some brain regions in this neural system are also engaged in the two other neural systems during aesthetic appreciation. Studies on meaning-knowledge neural system provide evidence that our aesthetic experience of art is influenced by the form of meaning and knowledge exert through a top–down processing [65, 66]. And positive emotion, induced by fluent conditions, has a possible causal effect on aesthetic preference [67]. According to the previous study, dorso-medial prefrontal cortex is a main area for social cognition and plays an important role in determine facial beauty and moral judgment [68]. The temporal pole and entorhinal cortex are activated when contextual information triggers memories and in turn modulates emotion [69]. The activation of precuneus is related to the way objects are labeled and the bilateral anterior cingulate cortex is activated when viewing preferred curves [70, 71]. These two brain regions, together with temporoparietal junction, show greater activation when viewing paintings under the Museum of Modern Art condition than under the adult center condition, indicating that these three brain regions are associated with distilling the semantic information from artworks [72]. According to another study contrasting aesthetic judgments and descriptive judgments on graphic patterns, the Brodmann areas 9/10 is associated with processing of internal information and shows enhanced activation when participants perform aesthetic judgments [56, 73].

Findings in previous neuroaesthetic studies provide evidence that cognitive processing of aesthetic appreciation recruits wildly distributed brain regions. Different brain region exhibits distinct functionality during aesthetic appreciation and the same brain region may play a different role in different processing stage. The parallel processing and co-working between different brain regions accomplish the process of aesthetic appreciation and constitute our aesthetic experience. To date, we still know litter about how these brain regions work together to form our aesthetic experience. Computational aesthetics and information theory may provide additional perspective and complement our understanding on aesthetic appreciation.

## Computational aesthetics on image quality assessment: mimic human’s aesthetic judgment using computer science

**Fig. 3:**
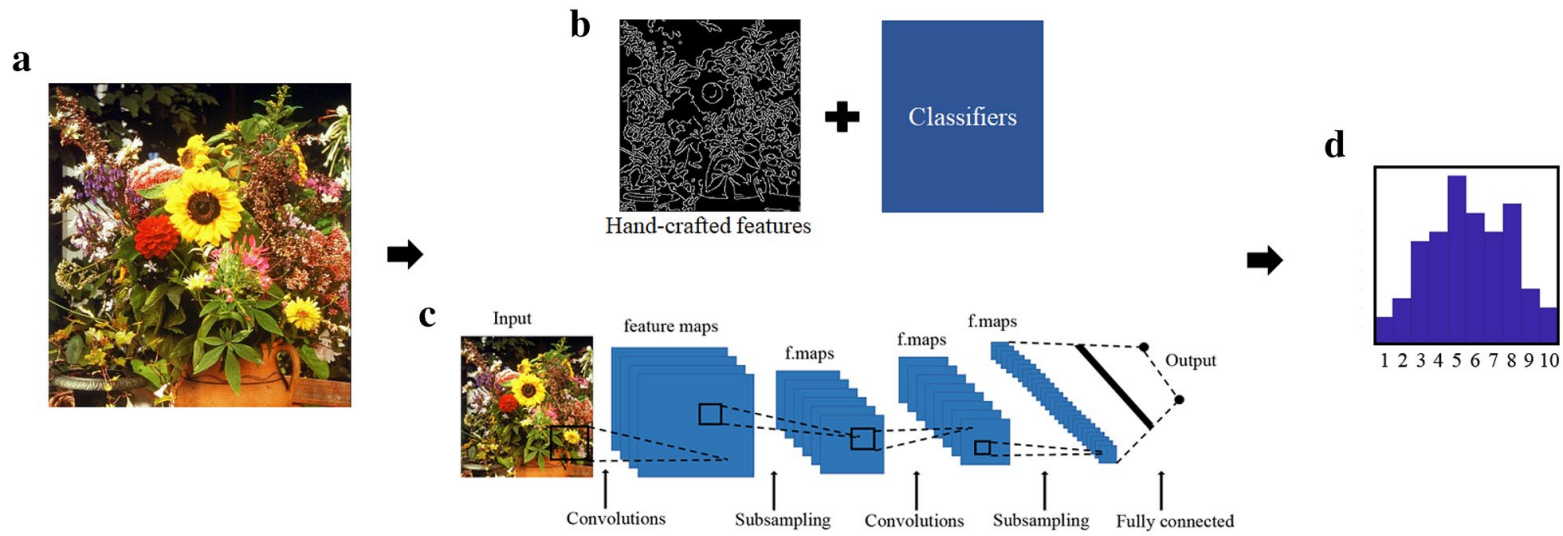
A workflow for image quality assessment. **a** The original image. **b** Hand-crafted features are extracted by computational algorithms and subsequently feed to classical classifiers. **c** Using neural network to automatically extract image features related to aesthetic ratings. **d** Final prediction

Computational aesthetics has emerged with the advance of digital technology and fast growth of computer science. Since the definition of computational aesthetics has been proposed by Hoenig in 2005, computational aesthetics grows vigorously in the past 15 years. The aim of computational aesthetics is to automatically and aesthetically evaluate visual objects like humans. One of the primary tasks in computational aesthetics is image quality assessment. In this task, new algorithms are developed to deal with extracting aesthetic features from images to make a judgment (see Fig. 3). Such algorithms have a wide application in our daily life like image recommendation system on website, artwork generation, and computer-aid photography.

### Image quality assessment based on hand-crafted features

Early studies on computational aesthetics mainly focused on image quality assessment based on hand-crafted features. These studies turned both experience from professional artists and existing knowledge of image properties from experimental aesthetics into formulas [74]. By designing a variety of hand-crafted formulas, these studies extracted low-level image features such as color, luminance, complexity, symmetry, etc. as well as high-level features such as spatial distribution of edges, Hue count, etc. from image dataset. Classifiers were constructed using machine learning algorithms such as support vector machines (SVM), naïve Bayes, and K-nearest neighbors (KNN). Then, constructed classifiers were trained by extracted image features as input and manual image quality assessment results as output to mimic human’s aesthetic evaluation. Finally, the whole computational models were used to achieve image quality assessment on new images by extracting image features using hand-crafted formulas and predicting aesthetic evaluation result with trained classifiers [75–78].

### Image quality assessment based on Deep Neural networks

In recent years, due to the outstanding performance of convolutional neural network (CNN) and deep learning on computer vision, studies on computational aesthetics paid more attention on how to construct deep neural networks to automatically learn image features from dataset than designing hand-crafted features. CNNs, which mimic visual processing of human eyes, use convolution kernel as receptive field to extract image features. Its parameters and prediction result are optimized by training with a loss function (e.g., the divergence between prediction result from neural network and human evaluation) on large image dataset [79]. The frequently used neural networks in current deep learning studies on computer vision are LeNet, AlexNet, GoogleNet, VGGNet, and ResNet [79–83]. In 2014, Wang et al. proposed a deep neural network named RAPID [84]. In their study, a double-column deep convolutional neural network (DCNN) was constructed and it took global and local information from images as input. Then, global and local image features were extracted and incorporated by DCNN to give an aesthetic evaluation. In addition, Wang et al. built a neural network called style-convolutional neural network (SCNN). This neural network extracted both image features and style attributes from images and integrated the two aspects to give an aesthetic evaluation. Both DCNN and SCNN achieved a good performance on a public aesthetic visual analysis dataset (AVA Dataset) [85]. Inspired by the neural mechanism and theoretical models of aesthetic appreciation, Dolcos et al. built a brain-inspired deep network [86]. This neural network contained several independent modules and processed input image by parallel pathways. Except the first three modules that computed simplest features (saturation, hue, and value), each module was a fully convolutional network and learned a selected feature dimension from image by a supervised training with individual labels. Then, image features extracted by these pre-trained modules were jointly feed to a high-level synthesis network. The high-level synthesis network integrated these features and gave a prediction on the distribution of aesthetic rating. In addition, Ge et al. improved the GoogleNet and proposed a neural network called ILGNet, which applied neural network architecture differ from DCNN to extract and combine local and global image features to predict aesthetic rating [87]. Other studies combined neural networks with different algorithms including auto-encoder technique, expert feature knowledge, feature fusion, visual attention, etc. to build new neural network architectures [88–92]. These neural networks leveraged distinct network architectures to automatically extract different image attributes and gave a prediction of aesthetic rating.

In computational aesthetics, to mimic human’s aesthetic evaluation on visual objects, numerous approaches of aesthetic measurement and neural network architectures were proposed. These researches enriched our understanding on the image attributes which an affect human’s aesthetic judgment. However, hand-crafted feature approaches only capture objective attributes of images, while neural networks with deep learning lack interpretability on extracted image features, which make it difficult for us to understand image features that can reflect people’s aesthetic evaluation.

## Computational neuroaesthetics: when computer science meets neuroaesthetics

New findings in neuroaesthetics provide insight for us on the neural mechanism of cognitive processing of aesthetic appreciation. And computational algorithms proposed in computational neuroaesthetics enrich our understanding on both objective attributes of images which can affect our aesthetic evaluation and the way we extract and process image features. Despite the flourishing of the two research fields, it seems that they are still independent from each other and evidence in one field cannot be directly applied or interpreted in another filed. This causes a huge gap between neuroaesthetics and computational aesthetics which hinders our comprehensive understanding on aesthetic appreciation. Computational neuroaesthetics seems to be a bridge to remedy the gap between these two fields. In computational neuroaesthetics, researchers analyzed neural activities from brain regions with computational algorithms to give a deep insight on how dynamic changes between brain regions form our aesthetic appreciation. Other studies, aiming at exploring features from neural activity related to our subjective aesthetic experience, applied computational algorithms on neuroimaging data to give a prediction on aesthetic preference.

### Brain functional connectivity and brain networks in neuroaesthetics: measuring the information flow during aesthetic appreciation

**Fig. 4:**
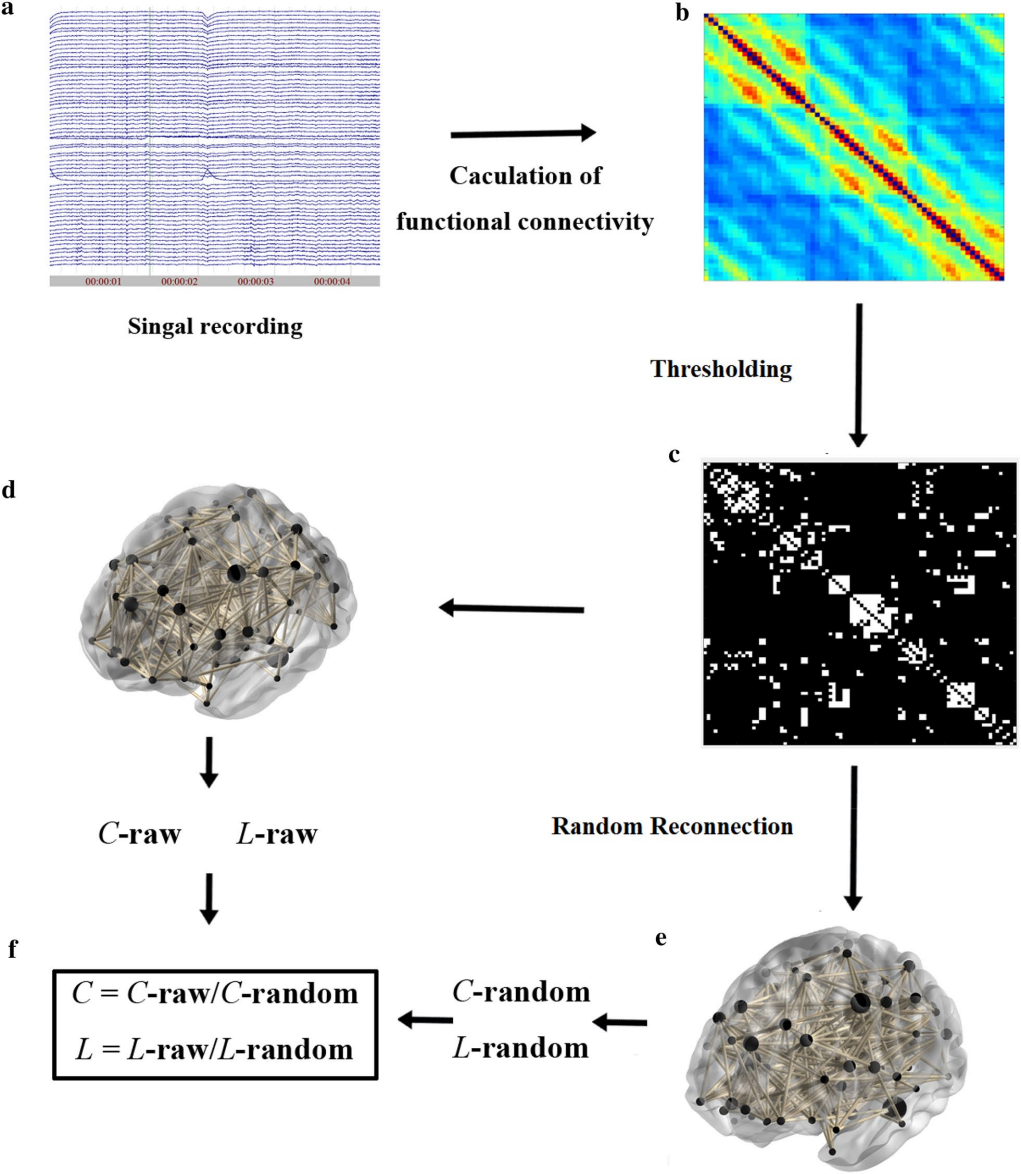
A workflow from functional connectivity measurement to brain functional network attributes. **a**. Neuroimaging data recorded from device, these data can be EEG, fMRI, or MEG. **b** Functional connectivity between brain regions is estimated from signals and form a correlation matrix. **c** The correlation matrix produces a binary connectivity graph by thresholding. **d** The visualization of binary graph. **e** The binary connectivity graph is randomly reconnected to produce a random reconnected graph. **f** The cluster coefficient (*C*) and average shortest path length (*L*) are extracted by measuring binary connectivity graph and random reconnected graph to obtain normalized brain functional network attributes

As we have mentioned above, aesthetic appreciation recruits activation of widely distributed brain regions and communication between these brain regions. Studies on brain activity tell us the relationship between specific brain region and aesthetic appreciation. However, the neural mechanism about how aesthetic appreciation emerges from the interaction between brain regions remains elusive. The interaction between brain regions cannot be directly observed from neuroimaging data. Functional connectivity is thought to be a useful measurement to provide a complementary understanding to the previous studies on brain activity [93]. It is a measurement of the information sharing and functional dependence between brain regions. Aiming at underlining the information flow between two brain regions, functional connectivity measures interaction between brain regions by applying computational algorithms on neuroimaging data recorded during cognitive processing (see Fig. 4). To date, the correlation analysis, the coherence, the phase locking value, the phase lag index, and the synchronization likelihood are frequently used to measure the functional connectivity. And the Granger causality is frequently used to measure the effective connectivity between brain regions.

Using the measurement of functional connectivity, previous studies found interactions between different brain regions during aesthetic processing. Brown reviewed previous neuroimaging studies on aesthetic appraisal in 2011 and proposed a functional connectivity model based on these studies [14]. In his model, the interaction between the anterior insula and the OFC plays an essential role in aesthetic processing and this connectivity is not restricted to aesthetic processing, but may be related to a more general cognitive processing—the assignment of valence to objects. Tsukiura and Cabeza studied memory encoding of attractiveness of faces and found that attractive faces were better remembered than other faces, with the increased activity of both the right OFC and the left hippocampus. Using the correlation analysis, they found that functional connectivity between these regions was stronger when encoding the beautiful faces, indicating that better memory is associated with greater interaction between reward and memory encoding [46]. Lacey et al. used event-related fMRI to study changes of brain activity between art images and non-art images. By applying Granger causality on fMRI data, they found that the ventral striatum was engaged in contemplation of art images [64]. Specifically, activity of ventral striatum was driven by visual cortex but not by brain regions related to aesthetic preference. In addition, using fMRI with dynamic casual modeling, Zhou et al. investigated the anticipation and evaluation of facial attractiveness [94]. They found bidirectional connectivity between the ventral striatum and the ventral medial prefrontal cortex existed during evaluation of attractive faces, but weakened for unattractive faces. This connectivity might reflect the dynamic process for the visual and aesthetic properties of faces. In 2018, Iwasaki studied the aesthetic perception of visual features by comparing sculptures obeyed the golden ration (canonical sculpture) with sculptures in which golden ratio was impaired (deformed sculpture) [95]. They found that the connectivity between the right occipital–temporal region and the right parietal region was correlated with the presentation of canonical sculptures but not for deformed sculptures, suggesting a neural pathway between these regions during the processing for aesthetic information.

In our brain, interactions between widely distributed brain regions and temporal variability of brain activity form a complex system [96, 97]. This complex system undergoes a dynamic reconfiguration during multiple cognitive processing stages of aesthetic appreciation [98–100]. To study such a complex system, brain functional networks, where nodes represent spatial location of brain regions and edges represent functional connections between these brain regions, are constructed from neuroimaging data. Once functional networks have been constructed, graph theory can provide helpful tools to measure properties of networks. Using graph theory, local integration which represents functional specific modules formed by connections between local brain regions, global integration which represents network ability to integrate distributed information between remote brain regions or modules, and the trade-off between activity cost and information process efficiency in the brain, can be represented by network properties named cluster coefficient, average shortest path length, and small world index, respectively [101]. Changes of these network properties can provide insights for us to study how our brain acts as a dynamic neural system during cognitive processing of aesthetic appreciation.

Using fMRI and Pearson correlation analysis, Lin et al. studied the effect of long-term artistic training on resting-state functional connectivity networks [102]. They found that the long-term artistic experience did not alter the communication efficiency, short-range and long-range connectedness, and modularity. However, actual modules, mainly in the bilateral cerebellum, showed significant difference between artistic professions and non-artists. This difference showed that even in the resting state, long-term training could imprint a neural network system in which brain regions are functionally and topologically modularized in domain-general as well as domain-specific manners. And results also suggested a resilient plasticity of our brain. In 2018, Pollick et al. also used fMRI to study aesthetic experience when processing diverse sensory input [103]. Using inter-subject correlation analysis, they identified several brain regions consistently activated when participants were watching a dance video accompanied by a soundtrack. These brain regions and their functional connectivity formed into a network which was composed of eight subnetworks. Six of the sub networks were related to the processing of sensory and motor aspects in observation. And the remaining two subnetworks appeared to be involved in complex cognitive activities. Specifically, one of the eight sub networks overlapped with the default mode network (DMN), which is considered being important in the access of internal information.

As these studies using fMRI are informative, the dynamic nature of aesthetic appreciation cannot be fully revealed by relatively slow temporal resolution of fMRI signals. Instead, MEG and EEG have relatively high temporal resolution and recently were used for brain functional network analysis of aesthetic appreciation. Cela-Conde et al. used MEG to acquire neuroimaging data when participant decided whether a stimulus was beautiful or not. Brain functional networks were constructed using phase locking value to analyze functional connectivity between different brain regions from MEG data [104]. They found aesthetic experience relied on two distinctive networks on the time course: (1) an initial network, where the OFC plays an important role, is associated with fast aesthetic perception; (2) another delayed network, which encompasses brain regions partially coincide with the DMN, is engaged during cognitive processing of beautiful stimuli and yields an aesthetic appreciation. Wu et al. used Chinese traditional music and EEG to study functional network changes during aesthetic appreciation [105]. They found that brain networks underwent a significant reconfiguration while participants were listening to music compared with noise and silence background. During the reconfiguration of brain networks in the alpha2 band, functional connections between the frontal–parietal and the temporal–parieto-occipital regions tend to induce an increasing synchronization and the whole network shifts to a more random structure. To investigate aesthetic experience in a realistic scene, Konston et al. used mobile EEG to record brain activity when subjects were freely moving and viewing exhibition at the Menil Collection in Houston [106]. Compared with viewing a blank wall, viewing the most aesthetic pleasing art elicited a significant increasing in connection strength between posterior and anterior areas in the delta and gamma bands.

Using computational approaches to measure the interaction between brain regions from mesoscale (cortical regions) as well as macroscale (brain scalp), previous studies investigated changes of functional connectivity as well as brain networks during aesthetic appreciation. These studies provided evidence on how our brain integrates sensory input with internal state to produce an aesthetic appreciation. Nevertheless, since this field is young, current studies only precisely identified the interaction between small portions of the whole brain. The interaction between a large proportion of brain regions from the three neural systems underlying aesthetic experience remains unclear. Much work still needs to be done and findings from mesoscale and macroscale should be integrated to give a fully understanding on the connectivity changes of the whole brain during distinct aesthetic processing stage.

### Machine learning in computational neuroaesthetics: aesthetic preference prediction based on neuroimaging data

**Fig. 5:**
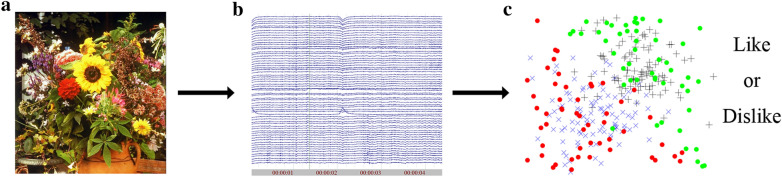
A workflow for aesthetic preference prediction based on neuroimaging data. **a** The original picture. **b** Neuroimaging data are recorded by EEG, MEG, or fMRI when participants are viewing images. **c** Bio-features are extracted from neuroimaging data and machine learning algorithms are applied to give a prediction of aesthetic preference

From the perspective of computational aesthetics, it is a hard challenge to assess aesthetic rating from individuals, since visual aesthetic objects only reflect objective attributes. Rating score from individuals is the subjective outcome of a series of complex cognitive processing on visual aesthetic objects. To capture subjectivity of aesthetic preference, neuroimaging data which can reveal brain activity during cognitive processing of aesthetic appreciation is indispensable. By applying machine learning algorithms on these neuroimaging data, we can mine bio-features which are associated with cognitive processing of aesthetic appreciation (see Fig. 5). Changes of these features on one hand provide additional information to assess subjectivity of aesthetic preference and on the other hand validate findings from neuroaesthetic studies.

To study aesthetic preference prediction on music, Stelios et al. collected EEG signals from subjects listening to music and used time–frequency analysis method to extract features from EEG data [107]. These features were further feed to KNN classifier to predict subject’s music preference. Their study achieved a good classification accuracy, and found that the beta and gamma bands played an important role in prediction of music preference. Chew and Teo investigated prediction of aesthetic preference on 3D shapes. The combination of time–frequency analysis and KNN classifier were applied on EEG signals recorded from subjects watching 3D shapes [108]. Features were extracted from the frontal region in the alpha, theta, and delta bands. Using these features with KNN, binary classification accuracy of 3D shapes preference can be obtained up to 80%. In 2018, Teo et al. recruited more participants to investigate prediction of aesthetic preference on 3D shapes [109]. This time, they applied a series of classifiers including SVM, random forest, Adaboost, KNN, and deep neural network to perform a binary classification. From the results, they found a combination of features from both widely distributed electrodes and frequency bands reached the highest accuracy. And deep neural network outperformed other machine learning classifiers.

In addition, Guo et al. recorded EEG and eye movement data when users were watching different styles of table lamp picture to predict user’s aesthetic preference [110]. The relative alpha power, relative gamma power, and average fixation time were extracted from the EEG and eye movement data. They found the average eye fixation duration was significantly different between low and high aesthetic lamps. And low aesthetic lamps evoked decreased relative alpha power accompanied by increased relative gamma power. By applying SVM, KNN, random forest, and XGboost classifiers, they achieved an accuracy of 82% for two-class classification (low versus high) and an accuracy of 73% for three-class classification (low versus middle versus high). Qu et al. used EEG to investigate prediction of aesthetic preference on Chinese typefaces [111]. Multiple EEG features were extracted from different frequency band and their correlation was analyzed by tensor multi-rank minimization. Then aesthetic preference of Chinese typefaces was predicted by multi-view self-representation clustering on selected features. They found that EEG data from different location of electrodes were correlated with accurate aesthetic preference prediction for different Chinese typeface. Particularly, central and parietal electrodes were correlated with disliked Chinese typefaces and frontal electrodes were correlated with liked Chinese typefaces.

Studies using machine learning algorithms on neuroimaging data could tell us which bio-feature is important to distinguish aesthetic preferred objects from others. And these features are helpful to understand neural activity related to aesthetic appreciation from the perspective of computer science. Nevertheless, these studies mainly used hand-crafted features and classic machine learning classifiers on EEG signals to predict aesthetic preference. Since neuroimaging data recorded from cognitive processing of aesthetic preference are varied on the time course, to capture bio-features from the neuroimaging data, new machine learning models which are suit for extracting features from time-varying signals, such as recurrent neural network (RNN), should be taken into consideration. And features designed based on empirical studies in neuroaesthetics such as brain functional connectivity can be helpful to improve the accuracy and provide theoretical support.

## Challenges and emerging prospects for the future of computational neuroaesthetics

In computational neuroaesthetics, several studies have been carried out to provide additional perspective for our understanding of aesthetic appreciation. As this field is still in its infancy, not enough data and findings exist to create a complete picture of how the beauty is processed to trigger an aesthetic outcome. To build an interdisciplinary research field which can bridge neuroaesthetics and computational aesthetics, much work still needs to be done and several issues should be addressed in computational neuroaesthetics.

### Measuring the dynamic information flow during aesthetic appreciation

To give a comprehensive understanding of aesthetic appreciation in computational neuroaesthetics, one challenge is to measure the dynamic information flow between precise brain regions during aesthetic appreciation. Aesthetic appreciation is influenced by multi-factors, including sensory attributes of visual objects such as complexity and symmetry, the way features of visual objects are represented in our perceptual system, context and embodiment of visual objects in our cognitive system, external context, internal state, top–down expectation, etc. [69, 112–116]. These factors alter connectivity between brain regions in the three neural systems during different processing stages of aesthetic appreciation.

To date, several studies have begun to scratch the surface on the impact from these factors, but they are still far from describing dynamic interactions between brain regions during aesthetic appreciation. For example, we still know litter about how the reward circuit triggered by dynamic projections from sensory and cognition system to create different types of appreciation and emotions. Moreover, during different processing stage of aesthetic appreciation, dynamic changes of the information flow between specific brain regions are still unknown and hard to be investigated. Low spatial resolution and volume conduction effect in EEG technology make it difficult to capture the information flow between precise brain regions. And low temporal resolution of fMRI cannot precisely reveal temporal changes of functional connectivity between brain regions. To measure the dynamic information flow between precise brain regions during aesthetic appreciation, multi-modal data need to be collected from both EEG and fMRI to reveal brain network changes with high temporal and spatial resolution. And more computational models should be developed to integrate brain connectivity changes from EEG and fMRI data and analyze how information flow will be affected by multi-factors.

### Predicting subjective rating of aesthetic appreciation: inter-group and intra-group difference

Another challenge is to predict subjective aesthetic rating of visual objects. Although studies in computational aesthetic can achieve a good performance when predicting aesthetic rating from a group of participants, these studies are not well enough to predict aesthetic rating from individuals. That is because when predicting aesthetic rating from individuals, both subjectivity and objectivity should be taken into consideration. Subjectivity has a close relationship with individual difference, which includes personality, culture, expertise, and observer’s sex. Individual difference is another important factor to influence aesthetic emotion and appreciation. It shapes the way we appreciate visual objects, produce unique aesthetic emotion, and determine outcomes of a subjective aesthetic appreciation. Thus, linking objectivity of visual objects with subjectivity of beholders is essential to give a precisely predicting on individual outcomes of aesthetic appreciation. And this is a crucial way to explore how multiple factors can influence our aesthetic appreciation.

It is hard to capture individual difference on aesthetic appreciation. Although several studies in neuroaesthetics have investigated influence of expertise, culture, and sex difference on aesthetic appreciation [117–121]. These studies are carried out by large samples and findings in these studies are uncovered mainly by statistical analysis based on inter-group comparison. Studies based on machine learning and neuroimaging data in computational neuroaesthetics will likely change this situation, but these studies are still coarse on extract features related to individual difference and subjective rating. To precisely predict aesthetic rating from individuals, one possible way is to find robust biomarkers related to subjective aesthetic appreciation. Previous studies predicting individual difference on creativity and attention may provide helpful ideas for us [122–126]. These studies performed correlation analysis on the relationship between activity of neural substrates and individual outcomes on creativity or attention. Changes of neural substrates which could robustly reflect individual outcomes were regarded as biomarkers and subsequently used to predict outcomes from new subjects. Recently, using the combination of machine learning and biomarkers, Vessel et al. investigated the domain-specific and domain-general coding of aesthetic appeal in the DMN and the ventral occipitotemporal cortex (VOT). fMRI data were collected when participants were making aesthetic judgments on images about art, architecture, and natural landscapes. Multi-voxel fMRI response patterns from the DMN and the VOT were subsequently used to train classifiers to predict participant’s aesthetic judgment (high versus low aesthetic appeal) from the same kind of photographs or from different kind of photographs. They found that activity pattern from the DMN was related to the prediction of aesthetic appeal across domains and extreme ratings to images were correlated with better predictions. Evidence from their study supports a model of aesthetic appreciation in which the DMN represents domain-general coding of visual aesthetic appeal [127].

## Conclusions

In conclusion, as an emerging interdisciplinary research field, computational neuroaesthetics plays an important role to bridge findings of neural substrates from neuroaesthetics and computation algorithms from computational aesthetics. Taking advantage from both methodological advances in computational aesthetics and theoretical advances in neuroaesthetics, computational neuroaesthetics can learn much from these two fields and gain increased momentum. We have seen that a series of recent works have ventured to study aesthetic appreciation from the point of machine learning and functional brain networks. Findings emerging from computational neuroaesthetics not only can increase our understanding on the neural mechanism of aesthetic appraisals, but also improve performance of computational models on predicting aesthetic rating. However, due to the complex processing and subjective nature of aesthetic appreciation, there are still difficulties and challenges exist in computational neuroaesthetics. The interaction of cognitive and emotional processing to produce aesthetic appreciation remains unclear and the computational architecture to mimic aesthetic appreciation still needs to be improved. As such, based on multi-modal data, a close tie between computational methodology and theoretical knowledge appears to be fruitful to advance our study in computational neuroaesthetics. Therefore, much work is necessary to employ new computational models such as Graph Neural Network to analyze brain connectivity and conceptualize changes of brain connectivity during aesthetic appreciation to improve computational models. A future direction relates to the integration of brain connectionist in neuroscience and neural network in machine learning may unravel how aesthetics emerge from the interaction of brain regions and build brain-inspired computational model which could truly mimic human’s aesthetic appreciation.

## Data Availability

Not applicable.

## Abbreviations

CNN: Convolutional neural network
DCNN: Double-column deep convolutional neural network
DLPFC: Dorsolateral prefrontal cortex
DMN: Default mode network
EEG: Electroencephalogram
FMRI: Functional magnetic resonance imaging
KNN: K-nearest neighbors
MEG: Magnetoencephalogram
OFC: Orbitofrontal cortex
PLV: Phase locking value
SCNN: Style-convolutional neural network
SVM: Support vector machine
VLPFC: Ventrolateral prefrontal cortex
VMPFC: Ventromedial prefrontal cortex
VOT: Ventral occipitotemporal cortex
